# Estimating the impact of tuberculosis anatomical classification on treatment outcomes: A patient and surveillance perspective analysis

**DOI:** 10.1371/journal.pone.0187585

**Published:** 2017-11-22

**Authors:** Otavio T. Ranzani, Laura C. Rodrigues, Eliseu A. Waldman, Carlos R. R. Carvalho

**Affiliations:** 1 Pulmonary Division, Heart Institute (InCor), Hospital das Clinicas (HCFMUSP), Faculdade de Medicina da Universidade de Sao Paulo, São Paulo, Brazil; 2 London School of Hygiene & Tropical Medicine (LSHTM), London, United Kingdom; 3 Department of Epidemiology, Faculty of Public Health, University of São Paulo, São Paulo, Brazil; Universidad Nacional de la Plata, ARGENTINA

## Abstract

**Introduction:**

Tuberculosis anatomical classification is inconsistent in the literature, which limits current tuberculosis knowledge and control. We aimed to evaluate whether tuberculosis classification impacts on treatment outcomes at patient and aggregate level.

**Methods:**

We analyzed adults from São Paulo State, Brazil with newly diagnosed tuberculosis from 2010–2013. We used an extended clinical classification of tuberculosis, categorizing cases as pulmonary, pulmonary and extrapulmonary, extrapulmonary and miliary/disseminated. Our primary outcome was unsuccessful outcome of treatment. To investigate the reported treatment outcome at the aggregate level, we sampled 500 different “countries” from the dataset and compared the impact of pulmonary and extrapulmonary classifications on the reported treatment success.

**Results:**

Of 62,178 patients, 49,999 (80.4%) were pulmonary, 9,026 (14.5%) extrapulmonary, 1,651 (2.7%) pulmonary-extrapulmonary and 1,502 (2.4%) miliary/disseminated. Pulmonary-extrapulmonary cases had similar unsuccessful outcome of treatment compared with pulmonary (adjusted-OR 1.00, 95%CI, 0.88–1.13, p = 0.941), while extrapulmonary were associated with better (adjusted-OR 0.65, 95%CI, 0.60–0.71, p<0.001) and miliary/disseminated with worse outcomes (adjusted-OR 1.51, 95%CI, 1.33–1.71, p<0.001). We found that 60 (12%) countries would report a difference ≥10% in treatment success depending on whether they reported all clinical forms together (current WHO recommendation) or pulmonary forms alone, overestimating the treatment success of pulmonary forms.

**Conclusions:**

The expanded anatomical classification of tuberculosis was strongly associated with treatment outcomes at the patient level. Remarkably, pulmonary with concomitant extrapulmonary forms had similar treatment outcomes compared with pulmonary forms after adjustment for potential confounders. At the aggregate level, reporting treatment success for all clinical forms together might hide differences in progress between pulmonary and extrapulmonary tuberculosis control.

## Introduction

Tuberculosis (TB) is an ancient disease still responsible for a high burden and high mortality worldwide[1]. TB can manifest in any tissue of the human body and the most common clinical presentation of active TB is pulmonary (PTB), representing, on average, around 80–85% of cases[1–5]. However, the proportion of extrapulmonary TB (EPTB) cases is sometimes higher in several low, middle and high-income countries[1, 2, 6, 7]: 43% in Cambodia[1] and 53% in England and Wales[3]. The differential distribution of some risk factors (e.g., aging and immunosuppression status) in each country is partly responsible for these discrepancies between PTB and EPTB burdens[4–6, 8]. Additionally, recent studies reported a relative increase in the proportion of EPTB cases, attributing it to the use of new immunosuppressive therapies, availability of diagnostic methods and better control of PTB[2, 3, 7, 9].

The definitions of PTB and EPTB are inconsistent in the literature[10]. Based mainly on the risk of transmission and guided by anatomy, the World Health Organization (WHO) classifies all patients into two categories: PTB (i.e., pulmonary, pulmonary with concomitant extrapulmonary, laryngeal and miliary disease) and EPTB (i.e., disease involving organs other than the lungs)[11]. However, other definitions from international societies and national organizations contain an extended anatomical TB classification and, additionally, report a third category labelled disseminated TB (i.e., miliary, two or more non-contiguous extrapulmonary sites or positive blood culture)[7, 12, 13]. This inconsistency in TB anatomical classification has direct implications for both the interpretation of TB epidemiology and program evaluations[4, 5, 7, 10], and for treatment decisions, with potential impact on treatment outcomes[13, 14].

The variations in EPTB classification constitute a public health problem that is neither widely recognized nor well understood. Although efforts have been made to improve diagnostic methods, EPTB has been considered a neglected disease[7, 10, 15]. We conducted this study with the primary aim of investigating whether an extended anatomical TB definition better identifies a patient’s likelihood of an unfavourable treatment outcome. Our secondary aim was to evaluate whether the definition impacts on the reported TB treatment outcome at the aggregate level as a public health perspective.

## Methods

### Study population

We conducted an observational retrospective analysis of routinely collected tuberculosis data from São Paulo State, Brazil on 62,178 patients with newly diagnosed TB from 2010 to 2013. São Paulo State has around 44 million inhabitants[16] and is responsible for the highest number (20%) of TB cases in Brazil, with an estimated TB incidence of 38 per 100,000 person years[17].

We included patients aged ≥15 years, newly diagnosed with TB (i.e. who had never been treated for TB or who had taken anti-TB drugs ≤1 month)[11] from 2010 to 2013. We excluded presumptive TB patients whose diagnosis had changed during the follow-up period and patients still on treatment at the time of database acquisition.

### Data source

We used data from the dedicated electronic system of the São Paulo State TB Program–“Tbweb”. This dedicated electronic platform includes all notified cases of bacteriologically or clinically confirmed cases of TB from residents in São Paulo State. TB notification is compulsory in Brazil and only notified cases can start treatment. Tbweb also receives continuous input regarding patient and treatment status from health-care units responsible for patient care. The platform has several steps for data quality, accuracy and consistency[17].

We had official written permission to use the data from the Data Guardians, the Health Department of São Paulo State and Ethical Approval from the local Ethics Committee (Comitê de Ética em Pesquisa da Faculdade de Medicina da Universidade de São Paulo—protocol 270/14).

### Definitions

Clinical TB form: the attending clinician determined the site of the disease according to a pre-specified list of sites (pulmonary, pleural, lymphatic, bone, genital, urinary tract, central nervous system, intestinal, ocular, skin, laryngeal, other, or multiple organs/miliar). The electronic system allowed the clinician to enter up to a maximum of three sites. Using the data available in the database, we derived two different clinical classifications (Table 1):

**Table 1:** Two clinical classifications of active tuberculosis.

	Classification 1 (WHO)	Classification 2
Pulmonary (lung parenchyma)	PTB	PTB only
Pulmonary and any concomitant extrapulmonary forms	PTB	PTB + EPTB
Miliary	PTB	Miliary/Disseminated
Laryngeal	PTB	EPTB only
Extrapulmonary – 1 organ affected	EPTB	EPTB only
Extrapulmonary – 2 or more non-contiguous organs affected	EPTB	Miliary/Disseminated

Abbreviations: EPTB—extrapulmonary tuberculosis; PTB—pulmonary tuberculosis; WHO—World Health Organization

Classification 1 (following the WHO classification) has two potential forms: 1) Pulmonary (i.e., lung parenchyma, pulmonary with any concomitant extrapulmonary site, laryngeal and miliary TB) and 2) Extrapulmonary (any other site occurrence and combinations)[11].

Classification 2 (following international societies’ classification) has four potential forms: 1) Pulmonary only, 2) Pulmonary and any concomitant extrapulmonary sites and combinations, 3) Extrapulmonary only and 4) Miliary/Disseminated (disseminated defined as occurrence of two or more sites not including lung parenchyma and/or a positive blood culture)[4, 5, 7, 10, 13, 18, 19].

Classification 2 was our exposure of interest, because we hypothesized that it better discriminates patients by their risk of worse outcomes.

Treatment outcomes: we used the 2013 WHO definitions, adapting them to the “TBweb”. This definition consists of 6 outcomes (Table A in S1 File), grouped into treatment success (“desired” outcomes) and unsuccessful treatment outcomes (“undesirable” outcomes). Treatment success is defined as the sum of “Cured” and “Treatment completed”. Briefly, “Cured” is defined for patients with PTB with bacteriologically confirmed TB at the beginning of treatment who had proven negative microbiological results upon treatment completion. “Treatment completed” is defined for patients with TB who completed treatment without evidence of clinical failure, but with no record to show negative microbiological results upon treatment completion, either because tests were not done or because no biological material was available (e.g., patient without sputum production). Unsuccessful outcomes comprised four possible outcomes: treatment failure, death, loss to follow-up and not evaluated[11, 17]. Full description of each outcome is described on the supplementary file (Table A in S1 File).

Potential confounders: we selected a priori potential confounders to be adjusted for, including those related to patients (age, sex, country of birth, self-reported race, homelessness, education level, alcohol and drug use, diabetes mellitus, mental disorder, HIV status, immunosuppression other than HIV) and those related to disease and treatment (place of diagnosis, chest x-ray and microbiologic status at diagnosis, initial drug scheme and directly observed-treatment—DOT). HIV status was classified as HIV negative, positive or unknown, as recommended by the WHO 2013 definitions[11].

### Data analysis

We described continuous variables with mean (SD) or median [IQR], depending on the variable distribution. Categorical variables were described as counts and percentage and compared using Fisher’s exact test or a chi-square test, as appropriate.

We used logistic regression models to evaluate the impact of the four different forms of TB disease (Classification 2) on the unsuccessful outcome of treatment (“primary outcome”) and death (“secondary outcome”) at patient level. First, we obtained crude odds ratios (OR) with 95% confidence interval (CI). We then obtained adjusted ORs (adjOR) allowing for all the potential confounding factors defined a priori in a multivariable logistic regression model. We evaluated a potential interaction between our exposure and HIV status in the final model[17]. Multicollinearity was assessed by the amount of variation on the standard errors of parameters on the logarithmic scale. To deal with missing data, we investigated the missingness patterns among variables and described the number of missing values and their associations (Tables B and C in S1 File). We assumed the missing values to be missing at random (MAR) and explored whether the missing values were conditioned on observed variables, suggesting a MAR mechanism. We did not expect a missing not at random pattern[20]. Our main analysis was based on five multiple imputed datasets. We also report the complete case analysis in a sensitivity analysis. For the patient level analysis, we excluded patients who were diagnosed upon necropsy, because our aim was to evaluate TB treatment outcomes[17, 21].

We conducted two additional analyses to investigate the impact of different TB classifications on the aggregate reported data at country level for a public health perspective. We generated 500 datasets with different sample sizes, simulating different countries, by sampling our data using bootstrapping, taking account of the four TB clinical presentations (Classification 2) and HIV status. In each dataset, we compared the difference in treatment success for each simulated country that would be reported for pulmonary and extrapulmonary TB between the two classifications (e.g., treatment success of PTB as defined in Classification 2 minus treatment success of PTB as defined by WHO). Finally, we compared the impact on treatment success at country level according to whether it was reported as currently recommended by WHO (treatment success of PTB + EPTB altogether) or as PTB and EPTB separately. In both analyses at country level, we reported the crude treatment success and the adjusted one by fitting the final multivariate model in each dataset.

We followed the STROBE guidelines and all analyses were conducted in STATA 13.1 (StataCorp-Texas).

## Results

Of 67,044 patients with newly diagnosed TB, we excluded 2,384 (3.6%) patients aged <15 years, 2,023 (3.0%) patients whose diagnosis had changed and 459 (0.7%) still on treatment. Finally, we analyzed 62,178 patients with newly diagnosed TB from 2010 to 2013 from São Paulo State.

### General characteristics of patients and clinical presentation

Most patients were classified as PTB only (n = 49,999; 80.4%), followed by EPTB only (n = 9,026; 14.5%). A further 1,651 (2.7%) patients had concomitant PTB-EPTB, while 1,502 (2.4%) had miliary/disseminated form (1,071–71.3% Miliary and 431–28.7% disseminated).

Table 2 describes the characteristics of patients over the four clinical presentations. Patients with PTB only were younger, less frequently white and had lower education levels. PTB only patients were mainly diagnosed in primary care, had higher microbiological confirmation and higher frequency of DOT. Patients with EPTB only had the highest female/male ratio, and lower prevalence of homelessness, alcohol and drug use. Miliary/disseminated cases were older, had higher prevalence of HIV positive status and other immunosuppression, and were more frequently diagnosed at necropsy. PTB-EPTB patients were nearly three times more likely to have HIV positive status or other immunosuppression than PTB only, and were mainly diagnosed during hospitalization.

**Table 2:** Comparison of general characteristics of patients newly diagnosed with tuberculosis classified in four clinical forms (n = 62,178).

Variable	Values	Pulmonary TB (n = 49,999)	Pulmonary and Extrapulmonary TB (n = 1,651)	Extrapulmonary TB (n = 9,026)	Miliary/ Disseminated TB (n = 1,502)	P value
**Age**, years	15–25	10,305 (20.6%)	249 (15.1%)	1,520 (16.9%)	153 (10.2%)	<0.001
	25.1–35	13,414 (26.9%)	436 (26.4%)	2,312 (25.6%)	350 (23.3%)	
	35.1–45	9,593 (19.2%)	384 (23.3%)	1,988 (22.0%)	432 (28.8%)	
	45.1–55	8,218 (16.5%)	292 (17.7%)	1,484 (16.5%)	278 (18.5%)	
	55.1–65	4,963 (9.9%)	175 (10.6%)	940 (10.4%)	161 (10.7%)	
	65.1–75	2,262 (4.5%)	79 (4.8%)	478 (5.3%)	75 (5.0%)	
	75.1–85	981 (2.0%)	32 (1.9%)	230 (2.6%)	44 (2.9%)	
	85.1–105	211 (0.4%)	4 (0.2%)	68 (0.8%)	7 (0.5%)	
	Missing	52 (0.1%)	-	6 (0.1%)	2 (0.1%)	
**Sex**	Female	13,935 (27.9%)	512 (31.0%)	3,634 (40.3%)	463 (30.8%)	<0.001
	Male	36,064 (72.1%)	1,139 (69.0%)	5,392 (59.7%)	1,039 (69.2%)	
**Country of birth**	Brazil	41,557 (97.3%)	1,370 (96.8%)	7,536 (97.7%)	1,211 (97.4%)	0.124
	Not Brazil	1,177 (2.7%)	45 (3.2%)	180 (2.3%)	32 (2.6%)	
	Missing	7,265 (14.5%)	236 (14.3%)	1,310 (14.5%)	259 (17.2%)	
**Self-reported race**	White	22,119 (51.0%)	841 (56.2%)	4,646 (58.1%)	794 (58.6%)	<0.001
	Black	5,085 (11.7%)	171 (11.4%)	872 (10.9%)	182 (13.4%)	
	Brown/Mixed	15,361 (35.4%)	459 (30.7%)	2,333 (29.2%)	363 (26.8%)	
	Asian	452 (1.0%)	21 (1.4%)	117 (1.5%)	12 (0.9%)	
	Indigenous	353 (0.8%)	5 (0.3%)	33 (0.4%)	5 (0.4%)	
	Missing	6,629 (13.3%)	154 (9.3%)	1,025 (11.4%)	146 (9.7%)	
**Education**	Illiterate	1,591 (3.9%)	41 (3.2%)	199 (2.8%)	39 (3.6%)	<0.001
	1–3 years	4,879 (12.1%)	122 (9.5%)	598 (8.3%)	116 (10.6%)	
	4–7 years	15,488 (38.4%)	428 (33.2%)	2,071 (28.6%)	404 (36.7%)	
	8–11 years	14,864 (36.8%)	540 (41.8%)	3,136 (43.3%)	417 (37.9%)	
	12–14 years	2,468 (6.1%)	110 (8.5%)	774 (10.7%)	67 (6.1%)	
	≥15 years	1,094 (2.7%)	50 (3.9%)	464 (6.4%)	57 (5.2%)	
	Missing	9,615 (19.2%)	360 (21.8%)	1,784 (19.8%)	402 (26.8%)	
**Homelessness**	No	48,616 (97.2%)	1,610 (97.5%)	8,962 (99.3%)	1,458 (97.1%)	<0.001
	Yes	1,383 (2.8%)	41 (2.5%)	64 (0.7%)	44 (2.9%)	
**Alcohol**	No	42,138 (84.3%)	1,395 (84.5%)	8,349 (92.5%)	1,281 (85.3%)	<0.001
	Yes	7,861 (15.7%)	256 (15.5%)	677 (7.5%)	221 (14.7%)	
**Diabetes mellitus**	No	46,858 (93.7%)	1,567 (94.9%)	8,617 (95.5%)	1,439 (95.8%)	<0.001
	Yes	3,141 (6.3%)	84 (5.1%)	409 (4.5%)	63 (4.2%)	
**Drug user**	No	44,459 (88.9%)	1,487 (90.1%)	8,609 (95.4%)	1,357 (90.3%)	<0.001
	Yes	5,540 (11.1%)	164 (9.9%)	417 (4.6%)	145 (9.7%)	
**Mental disorder**	No	49,061 (98.1%)	1,610 (97.5%)	8,871 (98.3%)	1,467 (97.7%)	0.102
	Yes	938 (1.9%)	41 (2.5%)	155 (1.7%)	35 (2.3%)	
**HIV status**	Negative	38,470 (76.9%)	993 (60.2%)	6,370 (70.6%)	684 (45.5%)	<0.001
	Positive	4,364 (8.7%)	461 (27.9%)	1,389 (15.4%)	633 (42.1%)	
	Unknown	7,165 (14.3%)	197 (11.9%)	1,267 (14.0%)	185 (12.3%)	
**Other immunosuppression**	No	49,616 (99.2%)	1,614 (97.8%)	8,875 (98.3%)	1,463 (97.4%)	<0.001
	Yes	383 (0.8%)	37 (2.2%)	151 (1.7%)	39 (2.6%)	
**Place of diagnosis**	PHC/Ambulatory	31,929 (64.8%)	568 (34.6%)	3,963 (44.6%)	420 (28.3%)	<0.001
	Emergency service	10,699 (21.7%)	385 (23.5%)	1,733 (19.5%)	338 (22.8%)	
	Hospital	6,148 (12.5%)	670 (40.9%)	3,104 (35.0%)	638 (43.0%)	
	Necropsy	512 (1.0%)	17 (1.0%)	79 (0.9%)	88 (5.9%)	
	Missing	711 (1.4%)	11 (0.7%)	147 (1.6%)	18 (1.2%)	
**Microbiological status**	Negative	6,790 (14.3%)	486 (32.8%)	4,218 (72.8%)	611 (51.0%)	<0.001
	Positive	40,861 (85.7%)	996 (67.2%)	1,580 (27.3%)	586 (49.0%)	
	Missing	1,836 (3.7%)	152 (9.3%)	3,149 (35.2%)	217 (15.4%)	
**Chest-X-ray**	Not done	7,782 (16.4%)	117 (7.4%)	1,572 (18.8%)	120 (8.9%)	<0.001
	Normal	1,718 (3.6%)	87 (5.5%)	2,342 (28.0%)	166 (12.4%)	
	Additional pathology	178 (0.4%)	24 (1.5%)	291 (3.5%)	30 (2.2%)	
	Suspect of TB	28,146 (59.5%)	1,154 (73.0%)	3,960 (47.3%)	928 (69.1%)	
	Suspect of TB + cavitation	9,503 (20.1%)	198 (12.5%)	206 (2.5%)	100 (7.4%)	
	Missing	2,160 (4.4%)	54 (3.3%)	576 (6.4%)	70 (5.0%)	
**Initial drug scheme**	Other	898 (1.8%)	59 (3.6%)	249 (2.8%)	58 (4.1%)	<0.001
	RHZ	489 (1.0%)	16 (1.0%)	111 (1.2%)	19 (1.3%)	
	RHZE	48,100 (97.2%)	1,559 (95.4%)	8,587 (96.0%)	1,337 (94.6%)	
**Directly observed treatment-DOT**	No	11,892 (24.1%)	733 (45.2%)	3,687 (41.5%)	622 (44.5%)	<0.001
	Yes	37,352 (75.9%)	890 (54.8%)	5,204 (58.5%)	775 (55.5%)	
	Missing	243 (0.5%)	11 (0.7%)	56 (0.6%)	17 (1.2%)	

Abbreviations: PHC–primary health care clinic; R–rifampicin; H–isoniazid; Z–pyrazinamide; E—ethambutol

### Site of the disease

**Fig 1:**
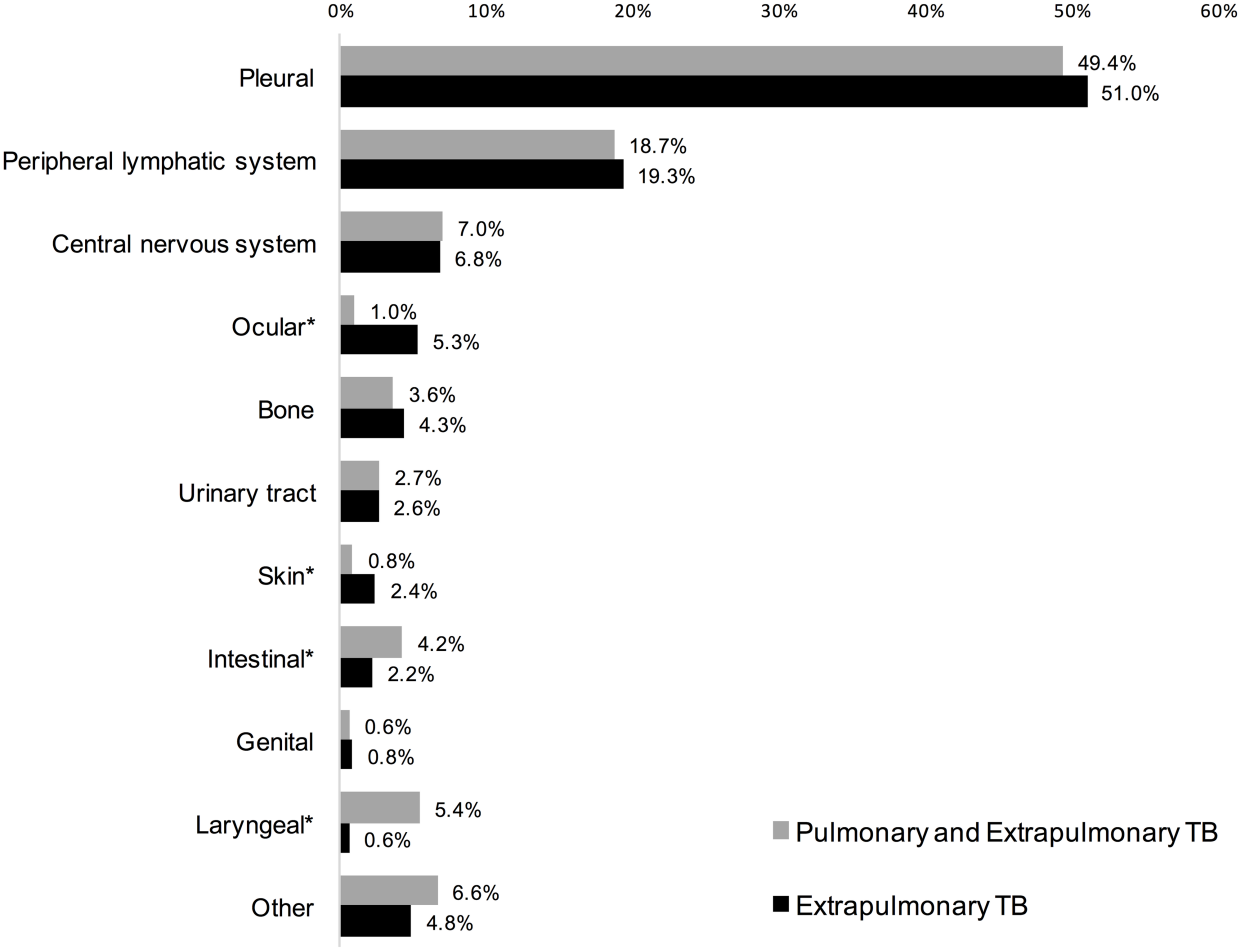
Prevalence distribution of extrapulmonary sites in patients with newly diagnosed tuberculosis presenting concurrent pulmonary or only extrapulmonary disease.

Pleural and lymphatic were the most common extrapulmonary TB sites, followed by central nervous system. Most sites were comparable among patients with PTB-EPTB and EPTB only. However, ocular and skin sites were more common in EPTB only compared with PTB-EPTB (5.3% vs. 1.0% and 2.4% vs. 0.8%, p<0.001, respectively). In contrast, intestinal and laryngeal sites were less common in EPTB only than PTB-EPTB (2.2% vs. 4.2% and 0.6% vs. 5.4%, p<0.001, respectively) (Fig 1).

### Treatment outcomes at patient level

The overall percentage of unsuccessful outcome of treatment was 18.9% (n = 11,744), mainly due to losses to follow-up (9.7%) and death (7.9%). EPTB only had the highest rate of treatment success (84.0%), followed by PTB only (81.5%), PTB-EPTB (73.2%) and miliary/disseminated (58.6%). Losses to follow-up were equally distributed (around 10%) among clinical forms, except for EPTB only at 7.5%. PTB-EPTB patients had two times higher mortality (14.2%) than PTB only and EPTB only, whereas miliary/disseminated had around four times higher mortality (30.8%). Table 3 shows the treatment outcomes for all patients stratified by different classifications.

**Table 3:** Treatment outcomes stratified by Classification 1 and 2 for newly diagnosed tuberculosis in São Paulo State, Brazil.

		Classification 1 (WHO)		Classification 2			
Outcomes	Overall (n = 62,178)	PTB (n = 52,773)	EPTB (n = 7,810)	PTB only (n = 49,999)	PTB-EPTB (n = 1,651)	EPTB only (n = 9,026)	Miliary/ Disseminated TB (n = 1,502)
Treatment success	50,434 (81.1%)	42,624 (80.8%)	7,810 (83.0%)	40,761 (81.5%)	1,208 (73.2%)	7,585 (84.0%)	880 (58.6%)
Unsuccessful outcome	11,744 (18.9%)	10,149 (19.2%)	1,595 (17.0%)	9,238 (18.5%)	443 (26.8%)	1,441 (16.0%)	622 (41.4%)
Treatment failure	306 (0.5%)	302 (0.6%)	4 (0.1%)	285 (0.6%)	13 (0.8%)	3 (0.1%)	5 (0.3%)
Death	4,925 (7.9%)	4,115 (7.8%)	810 (8.6%)	3,526 (7.1%)	235 (14.2%)	702 (7.8%)	462 (30.8%)
Loss to follow-up	6,003 (9.7%)	5,283 (10.0%)	720 (7.7%)	5,003 (10.0%)	176 (10.7%)	679 (7.5%)	145 (9.7%)
Not evaluated	510 (0.8%)	449 (0.9%)	61 (0.7%)	424 (0.9%)	19 (1.2%)	57 (0.6%)	10 (0.7%)

Abbreviations: EPTB—extrapulmonary tuberculosis; PTB—pulmonary tuberculosis; WHO—World Health Organization

The crude and adjusted associations between clinical forms and unsuccessful outcomes are shown in Table 4. EPTB only had lower odds of unsuccessful outcome of treatment and death compared with PTB only (adjOR 0.65, 95% CI, 0.60–0.71, p<0.001 and adjOR 0.59, 95% CI, 0.52–0.67, p<0.001, respectively). In contrast, miliary/disseminated were positively associated with unsuccessful outcome of treatment and death compared with PTB only (adjOR 1.51, 95% CI, 1.33–1.71, p<0.001 and adjOR 1.99, 95% CI, 1.72–2.31, p<0.001, respectively). However, PTB-EPTB had similar odds to unsuccessful outcome of treatment and death than PTB only after adjusting for potential confounders. Similar results were found in the complete case analysis (Table D in S1 File). We did not find an effect modification between the extended anatomical classification and HIV status.

**Table 4:** Crude and adjusted associations between clinical forms of TB and unsuccessful outcome of treatment and death (n = 62,178).

Variable	Unsuccessful outcome of treatment		Death	
**Crude**	**Crude OR (95% CI)**	**P value**	**Crude OR (95% CI)**	**P value**
Pulmonary TB	Reference		Reference	
Pulmonary and Extrapulmonary TB	1.65 (1.47–1.84)	<0.001	2.37 (2.05–2.75)	<0.001
Extrapulmonary TB	0.84 (0.79–0.89)	<0.001	1.15 (1.06–1.26)	0.002
Miliary/Disseminated TB	2.84 (2.54–3.16)	<0.001	5.55 (4.90–6.28)	<0.001
Adjusted*	**Adjust. OR (95% CI)**	**P value**	**Adjust OR (95% CI)**	**P value**
Pulmonary TB	Reference		Reference	
Pulmonary and Extrapulmonary TB	1.00 (0.88–1.13)	0.941	1.05 (0.89–1.24)	0.591
Extrapulmonary TB	0.65 (0.60–0.71)	<0.001	0.59 (0.52–0.67)	<0.001
Miliary/Disseminated TB	1.51 (1.33–1.71)	<0.001	1.99 (1.72–2.31)	<0.001

*Adjusted for age, sex, country of birth, race, education, homelessness, alcohol and drugs use, diabetes, mental disorder, HIV status, other immunosuppression, place of diagnosis, microbiologic diagnosis, Chest-X-Ray pattern at diagnosis, initial treatment drug and initial offer of directly observed treatment. Adjusted model from 5 multiple imputed datasets.

Abbreviations: CI—confidence intervals; OR—odds ratios; TB—tuberculosis.

### Treatment success at country-level

The average characteristics of the 500 samples (“500 countries”) are described in Table E in S1 File. The median sample size was 60,781 patients [36,447–88,689], with minimum 10,935 cases and maximum 165,641 cases. We found considerable variability in the distribution of the four clinical forms of TB and HIV status.

**Fig 2:**
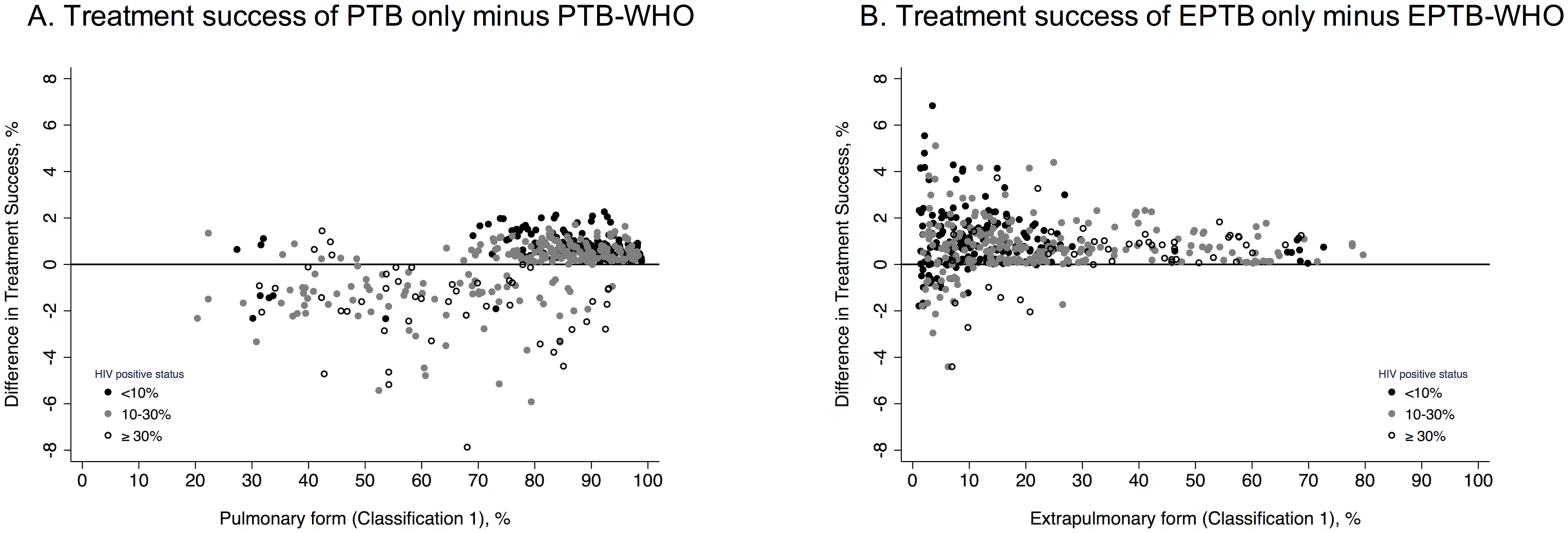
Over or underestimation in tuberculosis treatment success between the two clinical classifications at country level for 500 simulated countries. Panel A: difference in treatment success between Pulmonary TB only minus Pulmonary TB as classified by WHO (Classification 1); Panel B: difference in treatment success between Extrapulmonary TB only minus Extrapulmonary TB as classified by WHO (Classification 1).

The differences in treatment success that would be reported depending on whether each country reported PTB or EPTB only (Classification 2) instead of PTB or EPTB as Classification 1 are shown in Fig 2. Although the average differences would be around 1 or 2% (Fig 2; Table F in S1 File), for individual countries we can observe important differences. For instance, we observed 5 countries where the treatment success for “PTB only” would be over or underestimated by more than 5% (Table G in Si File). A country with 68% PTB and 32% EPTB (Classification 1) and high prevalence of HIV, would have reported a crude difference of 7.87% for PTB. Similarly, a country with 96.5% of PTB and 3.5% of EPTB (Classification 1) and low prevalence of HIV, would have reported a crude difference of 6.83% for EPTB (Fig 2, Table G in S1 File). The expected adjusted differences are shown in Fig A, Tables F and G in S1 File.

**Fig 3:**
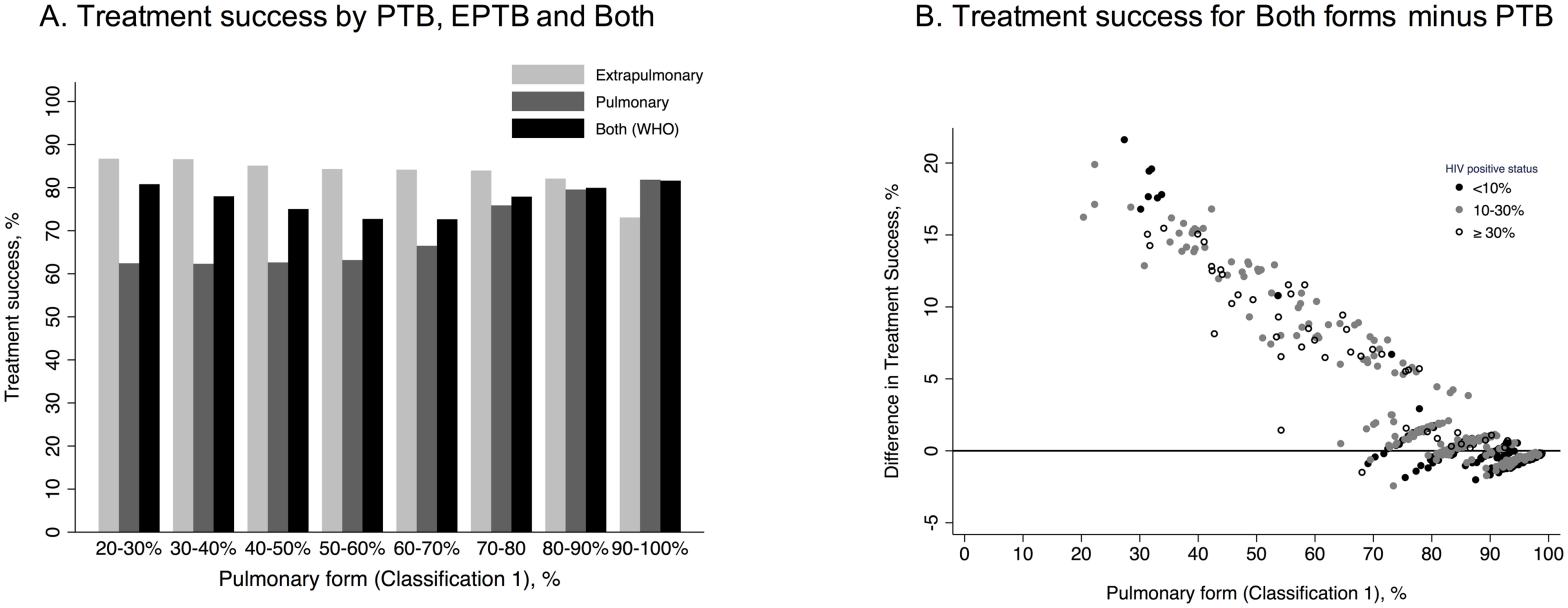
Over or underestimation in tuberculosis treatment success at country level for 500 simulated countries between overall (both forms) and each form of tuberculosis as classified by WHO (Classification 1). Panel A: Average treatment success for both forms, pulmonary and extrapulmonary tuberculosis; Panel B: difference in treatment success between overall (both forms) minus pulmonary TB as classified by WHO (Classification 1).

The differences in treatment success that would be reported if each country reports PTB and EPTB (Classification 1) separately instead of the overall rate between PTB and EPTB together are shown in Fig 3, Fig B and Table H in S1 File. We observed gaps between the treatment success achieved for each form (PTB or EPTB) and the treatment success of both forms together as evaluated by the WHO. Although the average gap was more evident for countries with a lower proportion of PTB, there were also huge gaps for individual countries with a high proportion of PTB. For instance, in a country with 60% PTB, the treatment success for both forms was 10% higher than that observed for PTB. There were 60 (12%) countries for PTB and 181 (36.2%) for EPTB where this gap would be more than 10% (Table I in S1 File). The expected adjusted differences are shown in Fig C, Tables H and I in S1 File.

## Discussion

We report three important findings regarding the impact of different clinical classifications of new TB cases. We demonstrated that the clinical classification had a strong association with treatment outcomes at the patient level. We showed that the difference in the reported treatment success at the country level would have a minor impact whether reporting PTB as Classification 1 (WHO) compared with PTB only as Classification 2. However, we showed that reporting the treatment success of both forms as currently recommended hides any difference in progress in PTB and EPTB. This has direct implications for national programs, because PTB is the main source of transmission, and in some countries the treatment success rate for PTB is lower than the overall success reported.

EPTB has rarely been studied; and when it is studied, the focus is on risk factors for EPTB[4, 5] and diagnostic methods[2], and few studies focused on treatment outcomes of EPTB[22]. One main barrier to interpreting previous studies is the lack of consistency between the clinical classifications used. Some reports excluded cases with pulmonary and concomitant extrapulmonary TB, others studied each form separately or mixed the disseminated forms into pulmonary or extrapulmonary classification[4, 8, 10]. In this study, we analyzed all forms classified in a proposed extended classification. Using this classification, we showed that after adjustments for potential confounders, patients with PTB only and PTB-EPTB had a very similar percentage of unsuccessful outcome of treatment and death. This was unexpected, requires replication and shows the importance of not excluding these patients from observational studies. In contrast, patients with EPTB only (mainly pleural and lymphatic disease) had better treatment outcomes. Current treatment guidelines have few evidence-based indications of customized treatment for different forms of TB, and it is likely that approaching TB treatment, taking into account its clinical presentation, will have an impact on treatment decisions and outcomes[13, 23, 24].

Miliary presentation is a classical form of TB defined by diffuse miliar infiltration on chest-X-ray and is attributed to a massive lymphohaematogeneous dissemination of the bacilli[25]. Although some classifications and clinicians still focus on the chest-X-ray pattern to classify miliary TB, it is well known that chest-X-ray has low sensitivity to diagnose diffuse micronodules[25, 26]. In addition, other forms of disseminated TB (also labelled bacteraemic, cryptic or generalized) have similar pathophysiology of miliary TB, with challenging diagnosis and paucity of symptoms, leading to delayed treatment and worse outcomes[25, 27, 28]. We showed that although a great part of the association between miliary/disseminated TB and worse outcomes was due to confounding factors, disseminated forms were still associated with unsuccessful outcome of treatment and death. We believe it should be a priority to treat disseminated forms of TB separately in official reports, guidelines and clinical studies, due to their particular features and worse outcomes.

The extended anatomical classification is likely to be applicable in clinical practice because it is simple and based on normally collected data in National TB Programs. However, it does not encompass other important features present in the complex pathogen-host interaction[29]. Indeed, the classification does not take account markers of immune response, or additional characteristics such as bacillary burden, and radiological pattern for pulmonary cases[29, 30]. Similarly, HIV positive patients deserve special attention, because of their expected worse outcomes and their TB clinical presentation, that could be unusual, limiting our extended “classical” anatomic classification. We did not observe an effect modification for HIV status, however we have not data about HIV stage (e.g., CD4 count and viral load) and antiretroviral therapy[31]. We believe that taking into account all these other important features, as well as using data-driven techniques in our “Big data era” (e.g., latent-variable analysis or machine learning)[32], a new classification based on clusters and phenotypes might show better performance compared with the extended anatomical classification.

TB remains a major public health problem and, although improvements have been observed in recent decades, the goals regarding TB control have not been achieved[1, 33]. In particular, several countries, including Brazil, did not achieve the expected treatment success rate of 85% recommended by WHO[1]. Since PTB is the main source of transmission, national TB programs should ensure that targets are monitored for these patients to decrease the risk of transmission. We hypothesized that reporting the overall treatment success (PTB+EPTB), as requested by the WHO, could hinder accurate monitoring of PTB, thereby distorting the evaluation of national programs. Our results clearly showed that both over and underestimation of treatment success for PTB and EPTB can happen in several scenarios when using the WHO classification at the aggregate level. Although some of the simulated scenarios are unlikely to occur (such as countries with less than 40% of PTB), a substantial percentage of countries could be reporting ≥10% difference in treatment success rates for PTB. Importantly, we showed that we have small changes in treatment success if reporting PTB only (Classification 2) instead of PTB as in Classification 1. The difference is expected to be small as PTB only is the major form of PTB in Classification 1, while other forms contribute very few cases, and poor outcomes occurred mainly in miliary disease, which corresponds to less than 2% of cases. WHO leaves the decision of whether to report treatment outcomes separately for PTB and EPTB to national programs[34].

We suggest, based on our results, that WHO should require all countries to analyze and report treatment outcomes separately for PTB and for EPTB. Indeed, the Centers for Disease Control and Prevention (CDC), the European Centre for Disease Prevention and Control (ECDC) and some national programs (e.g., Public Health England) already report TB epidemiology using an approach similar to the extended classification (Classification 2)[12, 19].

Our study used population-representative data from a high-burden country. We overcame previous literature limitations by including in our analysis the four clinical forms of TB. Additionally, we showed that reports and audits from national programs could be improved by separating treatment outcomes into TB forms. However, our study has some limitations. We included only new cases of TB in order to analyze a homogeneous population, but WHO considers both relapses and new cases to evaluate treatment outcomes. Further studies should assess the association of the extended anatomical classification and treatment outcomes in different settings, and not only in new cases, but also including relapses. Second, we used the standardized treatment outcomes as suggested by the WHO, but previous studies showed the impact on treatment success rate using different treatment outcomes classification[21]. The impact of the extended anatomical classification on treatment outcomes should be evaluated using other treatment outcomes definitions. Indeed, there is a gap on how to appropriately evaluate treatment outcomes for EPTB, and further research should focus on it, for a feasible and comparable treatment outcome definitions. Third, we excluded TB cases in children, a population where disseminated TB has an important impact on treatment outcomes. Fourth, we did not evaluate the treatment outcome for the EPTB group stratifying by each site (e.g., renal, adrenal, central nervous system). The fact that some sites have particular natural courses, such as long quiescent times, might explain in part our findings for better outcomes for EPTB. Finally, our main analysis was based on multiple imputation assuming a missing at random mechanism, that is an untestable assumption. However, we have not indications of missing not at random mechanism, based on the missingness pattern and on the researcher’s experience with the São Paulo TB database. Importantly, our results remained similar in our sensitivity analysis.

In summary, we observed that the anatomical classification of TB was strongly associated with treatment outcomes. We hope our results will encourage a discussion of how to better address the classification of TB for individual treatment and for public health reporting by the international community responsible for establishing policies, guidelines recommendations, research and for monitoring TB indicators.

## Supporting information

S1 File (PDF)
