# Supplementary material for: Estimating the impact of tuberculosis anatomical classification on treatment outcomes: A patient and surveillance perspective analysis
Source: PLoS One. 2017 Nov 22;12(11):e0187585. doi: 10.1371/journal.pone.0187585 (PMC5699807; doi:10.1371/journal.pone.0187585)
Supplement: S1 File — (PDF) [file pone.0187585.s001.pdf]

Supplementary material

Estimating the impact of tuberculosis anatomical classification on treatment outcomes: a patient and population perspective analysis

Otavio T. Ranzani, MD,
Prof. Laura C. Rodrigues, PhD,
Prof. Eliseu A. Waldman, PhD,
Prof. Carlos R. R. Carvalho, PhD

Table of Contents

Table A. Adapted TB Outcomes from WHO (not includes resistant TB) to the São Paulo State TB Program, Brazil[1] 2

Table B. Variables with missing values from the 61,482 patients included in the multivariate analysis after multiple imputation (i.e., excluding those diagnosed after death without previous diagnosis) 3

Table C. Missingness pattern evaluation 4

Table D. Crude and adjusted associations between clinical forms of TB and unsuccessful outcome of treatment and death in complete-case analysis 5

Table E. General characteristics of 500 samples ("500 country profiles") 6

Table F. Differences in the reported Treatment success rate at country level between Classification 1 and Classification 2 for Pulmonary and Extrapulmonary forms (n=500, simulated data) 7

Table G. Differences more than 2.5, 5 and 10% in the reported Treatment success rate at country level between Classification 1 and Classification 2 for Pulmonary and Extrapulmonary forms (n=500, simulated data) 7

Figure A. Over or under-estimation in tuberculosis treatment success at country-level for 500 simulated countries between the two clinical classifications after full adjustment for potential confounders. 8

Table H. Differences in the reported Treatment success rate at country level between Classification 1 and Classification 2 for Pulmonary and Extrapulmonary forms (n=500, simulated data) 9

Table I. Differences more than 2.5, 5 and 10% in the reported Treatment success rate at country level between Classification 1 and Classification 2 for Pulmonary and Extrapulmonary forms (n=500, simulated data) 9

Figure B. Over or under-estimation in tuberculosis treatment success at country-level for 500 simulated countries between overall (both forms) and extrapulmonary tuberculosis as classified by WHO (Classification1) 10

Figure C. Over or under-estimation in tuberculosis treatment success at country-level for 500 simulated countries between overall (both forms) and each for of tuberculosis as classified by WHO (Classification1) after full adjustment for potential confounders 11

Table A. **Adapted TB Outcomes from WHO (not includes resistant TB) to the São Paulo State TB Program, Brazil[1]**

**Outcome**	**Definition**
Cured	A pulmonary TB patient with bacteriologically confirmed TB at the beginning of treatment who was smear- or culture-negative in the last month of treatment and on at least one previous occasion
Completed treatment	A TB patient who completed treatment without evidence of failure but with no record to show that sputum smear or culture results in the last month of treatment and on at least one previous occasion were negative, either because tests were not done or because results are unavailable
Treatment failed	For the São Paulo State TB-Program, treatment failure was defined as any proven resistance to TB drugs during the treatment.
Died	A TB patient who dies for any reason before starting or during the course of treatment
Lost to follow-up	A TB patient who did not start treatment or whose treatment was interrupted for 2 consecutive months or more
Not evaluated	A TB patient for whom no treatment outcome is assigned. This includes cases “transferred out” to another treatment unit as well as cases for whom the treatment outcome is unknown to the reporting unit
Treatment success	The sum of cured and treatment completed
Cohort	A group of patients in whom TB has been diagnosed.<br>This group forms the denominator for calculating treatment outcomes. The sum of the above treatment outcomes, plus any cases for whom no outcome is recorded (including those ‘still on treatment’) and “transferred out” cases should equal the number of cases registered. Some countries monitor outcomes among cohort defined by sputum smear and/or culture, and define cure and failure according to the best laboratory evidence available for each patient.

1. World Health Organization-WHO. Definitions and reporting framework for tuberculosis - 2013 revision (updated December 2014). WHO Publications. 2013:1-47.

Table B. **Variables with missing values from the 61,482 patients included in the multivariate analysis after multiple imputation (i.e., excluding those diagnosed after death without previous diagnosis)**

Variable	Number of missing values	Percentage
Age	48	<0.1%
Directly observed treatment-DOT	327	0.5%
Place of diagnosis	887	1.4%
Chest-X-ray	2860	4.7%
Microbiologic status	5354	8.7%
Race	7815	12.7%
Country of birth	8873	14.4%
Education	11857	19.3%

Table C. **Missingness pattern evaluation**

Percent	Age	DOT	Place	Chest-X-ray	Microbiologic status	Race	Country of birth	Education
**Complete case analysis**								
59%	1	1	1	1	1	1	1	1
**Missing unique values**								
8%	1	1	1	1	1	1	1	0
5%	1	1	1	1	1	1	0	1
4%	1	1	1	1	1	0	1	1
5%	1	1	1	1	0	1	1	1
2%	1	1	1	0	1	1	1	1
<1%	1	1	0	1	1	1	1	1
<1%	1	0	1	1	1	1	1	1
<1%	0	1	1	1	1	1	1	1
**Combination of two simultaneously**								
3%	1	1	1	1	1	1	0	0
2%	1	1	1	1	1	0	1	0
1%	1	1	1	1	1	0	0	1
<1%	1	1	1	1	0	1	1	0
<1%	1	1	1	1	0	1	0	1
<1%	1	1	1	1	0	0	1	1
<1%	1	1	1	0	1	1	1	0
<1%	1	1	1	0	1	1	0	1
<1%	1	1	1	0	1	0	1	1
<1%	1	1	1	0	0	1	1	1
<1%	1	1	0	1	1	1	1	0
<1%	1	1	0	1	1	1	0	1
<1%	1	1	0	1	1	0	1	1
<1%	1	1	0	1	0	1	1	1
<1%	1	1	0	0	1	1	1	1
<1%	1	0	1	1	1	1	1	0
<1%	1	0	1	1	1	1	0	1
<1%	1	0	1	1	1	0	1	1
<1%	1	0	1	1	0	1	1	1
<1%	1	0	1	0	1	1	1	1
<1%	0	1	1	1	1	1	1	0
<1%	0	1	1	1	1	1	0	1
<1%	0	1	1	1	1	0	1	1
<1%	0	1	1	1	0	1	1	1
<1%	0	1	1	0	1	1	1	1
**Combination of three simultaneously**								
2%	1	1	1	1	1	0	0	0

All other combinations were <1%, reaching a maximum of 6 variables with missing values simultaneously (combination of ≥4: 509/0.83%)

DOT-Directly observed treatment

Table D. **Crude and adjusted associations between clinical forms of TB and unsuccessful outcome of treatment and death in complete-case analysis**

Variable	Unsuccessful outcome of treatment		Death	
**Crude (n=62,178)**	**Crude OR (95% CI)**	**P value**	**Crude OR (95% CI)**	**P value**
Pulmonary TB	Reference		Reference	
Pulmonary and Extrapulmonary TB	1.65 (1.47-1.84)	<0.001	2.37 (2.05-2.75)	<0.001
Extrapulmonary TB	0.84 (0.79-0.89)	<0.001	1.15 (1.06-1.26)	0.002
Miliary/Disseminated TB	2.84 (2.54-3.16)	<0.001	5.55 (4.90-6.28)	<0.001
**Adjusted* (n=36,347)**	**Adjust. OR (95% CI)**	**P value**	**Adjust OR (95% CI)**	**P value**
Pulmonary TB	Reference		Reference	
Pulmonary and Extrapulmonary TB	1.05 (0.89-1.24)	0.576	1.10 (0.87-1.39)	0.431
Extrapulmonary TB	0.66 (0.58-0.75)	<0.001	0.54 (0.44-0.65)	<0.001
Miliary/Disseminated TB	1.36 (1.13-1.63)	0.001	1.60 (1.27-2.01)	<0.001

*Adjusted for age, sex, country of birth, race, education, homelessness, alcohol and drugs use, diabetes, mental disorder, HIV status, other immunosuppression, place of diagnosis, microbiologic diagnosis, Chest-X-Ray pattern at diagnosis, initial treatment and initial offer of directly observed treatment. Adjusted model from 5 multiple imputed datasets.

CI denotes confidence intervals; OR denotes odds ratios; TB denotes tuberculosis.

Table E. **General characteristics of 500 samples ("500 country profiles")**

	Mean (SD)	Median [IQR]	p10%-p90%	Min-Max
**Sample size**	64,289 (31,464)	60,771 [36,173-89,121]	25,861 – 104,468	10,935 – 165,641
**Clinical forms distribution**				
**Classification 1 (WHO)**				
Pulmonary form	79.6% (18)	85.5% [73.5-92.5%]	49.4% - 97.7%	20.4% - 98.9%
Extrapulmonary form	20.4% (18)	14.5% [7.5-26.5%)	3.4 – 50.6%	1.1 – 79.7%
**Expanded classification**				
Pulmonary only	73.2% (21)	80.5% [64.4-88.2%]	37.4 – 93.1%	14.2 – 98.3%
PTB + ETPB	3.8% (4)	2.7% [1.2-4.8%]	0.4 – 7.8%	0 – 32.9%
EPTB only	19.5% (18)	13.6% [7.0-25.4%]	2.7 – 48.6%	1 – 79.3%
Miliar/Disseminated	3.4% (4)	2.2% [1.2-4.3%]	0.6 – 8.0%	0 – 29.4%
**HIV status distribution**				
Negative	67.2% (21)	73.4% [55.4-83.0%]	31.4 – 88.8%	9.7 – 96.8%
Positive	14.6% (11)	11.1% [6.9-18.3%]	4.6 – 30.2%	1.8 – 73.4%
Unknown	18.2% (15)	14.1% [6.8-24.6%]	3.1-42.4%	0.6-70.9%

EPTB denotes extrapulmonary tuberculosis; HIV denotes human immunodeficiency virus; PTB denotes pulmonary tuberculosis; WHO denotes World Health Organization.

Table F. **Differences in the reported Treatment success rate at country level between Classification 1 and Classification 2 for Pulmonary and Extrapulmonary forms (n=500, simulated data)**

	Mean (SD)	Median [IQR]	p10% - p90%	Min-Max
Comparing PTB only minus PTB-WHO				
Treatment success				
Crude	0.02% (1.4)	0.42% [-0.42 to 0.75%]	-1.76 to 1.17%	-7.87 to 2.27%
Fully adjusted	-0.35% (1.4)	0.23% [-0.93 to 0.41%]	-2.29 to 0.66%	-7.54 to 1.28%
Comparing EPTB only minus EPTB-WHO				
Treatment success				
Crude	0.85% (1.2)	0.75% [0.18 to 1.37%]	-0.03 to 2.17%	-4.41 to 6.83%
Fully adjusted	0.90% (1.2)	0.70% [0.20 to 1.36%]	-0.01 to 2.06%	-2.65 to 7.94%

EPTB denotes extrapulmonary tuberculosis; PTB denotes pulmonary tuberculosis; WHO denotes World Health Organization.

Table G. **Differences more than 2.5, 5 and 10% in the reported Treatment success rate at country level between Classification 1 and Classification 2 for Pulmonary and Extrapulmonary forms (n=500, simulated data)**

	Difference ≥ 2.5%	Difference ≥ 5%	Difference ≥ 10%
Comparing PTB only minus PTB-WHO			
Treatment success			
Crude	24 (4.8%)	5 (1.0%)	0 (0.0%)
Fully adjusted	42 (8.4%)	6 (1.2%)	0 (0.0%)
Comparing EPTB only minus EPTB-WHO			
Treatment success			
Crude	33 (6.6%)	3 (0.6%)	0 (0.0%)
Fully adjusted	38 (7.6%)	6 (1.2%)	0 (0.0%)

EPTB denotes extrapulmonary tuberculosis; PTB denotes pulmonary tuberculosis; WHO denotes World Health Organization.

Figure A. **Over or under-estimation in tuberculosis treatment success at country-level for 500 simulated countries between the two clinical classifications after full adjustment for potential confounders.**

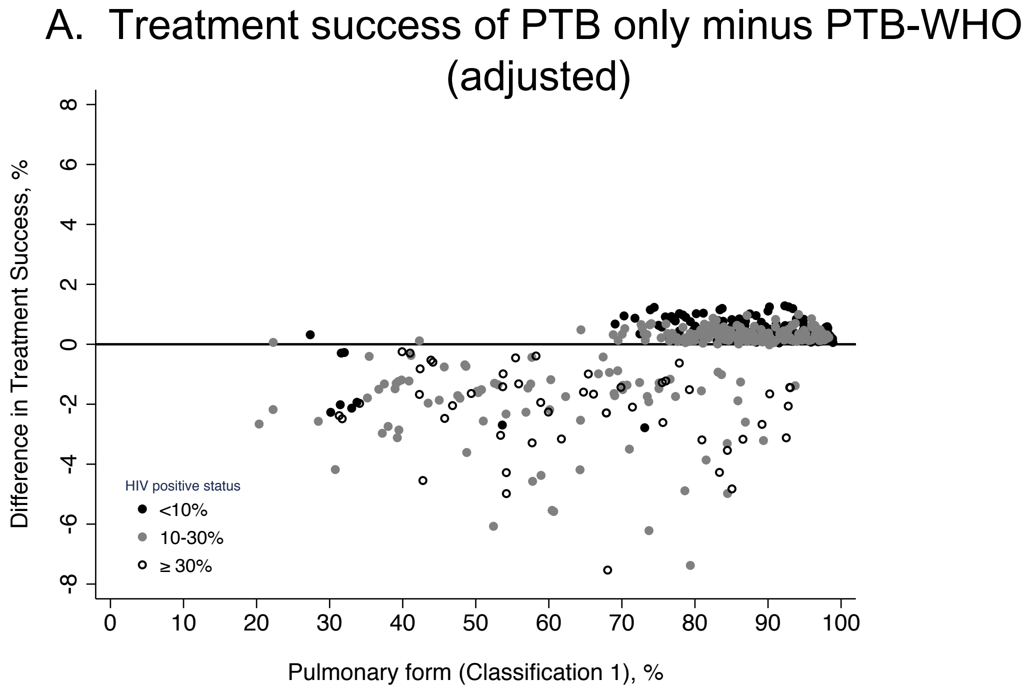

Panel A: difference in treatment success between Pulmonary TB only minus Pulmonary TB as classified by WHO (Classification 1)

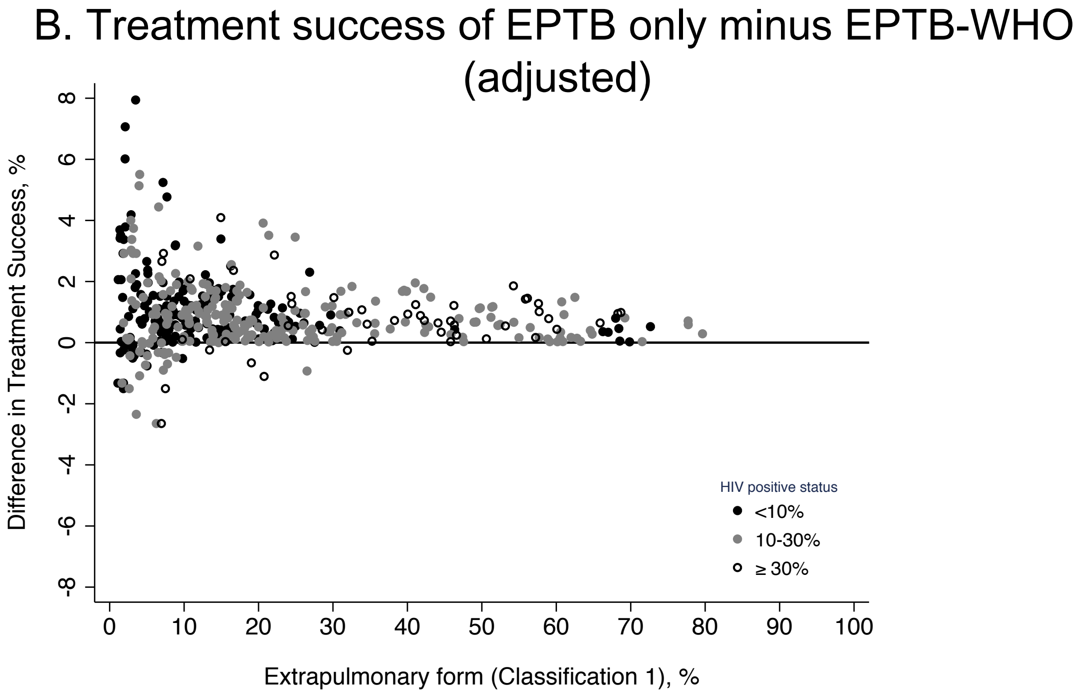

Panel B: difference in treatment success between Extrapulmonary TB only minus Extrapulmonary TB as classified by WHO (Classification 1).

Table H. **Differences in the reported Treatment success rate at country level between Classification 1 and Classification 2 for Pulmonary and Extrapulmonary forms (n=500, simulated data)**

	Mean (SD)	Median [IQR]	p10% - p90%	Min-Max
Comparing Both (PTB+EPTB) minus PTB				
Treatment success				
Crude	2.62% (5.1)	0.33% [-0.33 to 2.02%]	-0.82 to 12.00%	-2.44 to 21.62%
Fully adjusted	2.25% (4.3)	0.34% [-0.26 to 1.63%]	-0.61 to 9.70%	-1.62 to 18.88%
Comparing Both (PTB+EPTB) minus EPTB				
Treatment success				
Crude	-0.63% (9.9)	-1.84% [-8.05 to 6.74%]	-12.48 to 14.03%	-24.08 to 22.95%
Fully adjusted	-0.80% (8.1)	-1.87% [-6.49 to 5.93%]	-10.59 to 11.74%	-20.29 to 18.93%

EPTB denotes extrapulmonary tuberculosis; PTB denotes pulmonary tuberculosis; WHO denotes World Health Organization.

Table I. **Differences more than 2.5, 5 and 10% in the reported Treatment success rate at country level between Classification 1 and Classification 2 for Pulmonary and Extrapulmonary forms (n=500, simulated data)**

	Difference ≥ 2.5%	Difference ≥ 5%	Difference ≥ 10%
Comparing Both (PTB+EPTB) minus PTB			
Treatment success			
Crude	115 (23.0%)	110 (22.0%)	60 (12.0%)
Fully adjusted	114 (22.8%)	103 (20.6%)	45 (9.0%)
Comparing Both (PTB+EPTB) minus EPTB			
Treatment success			
Crude	374 (74.8%)	299 (59.8%)	181 (36.2%)
Fully adjusted	354 (70.8%)	271 (54.2%)	129 (25.8%)

EPTB denotes extrapulmonary tuberculosis; PTB denotes pulmonary tuberculosis; WHO denotes World Health Organization.

Figure B. **Over or under-estimation in tuberculosis treatment success at country-level for 500 simulated countries between overall (both forms) and extrapulmonary tuberculosis as classified by WHO (Classification1)**

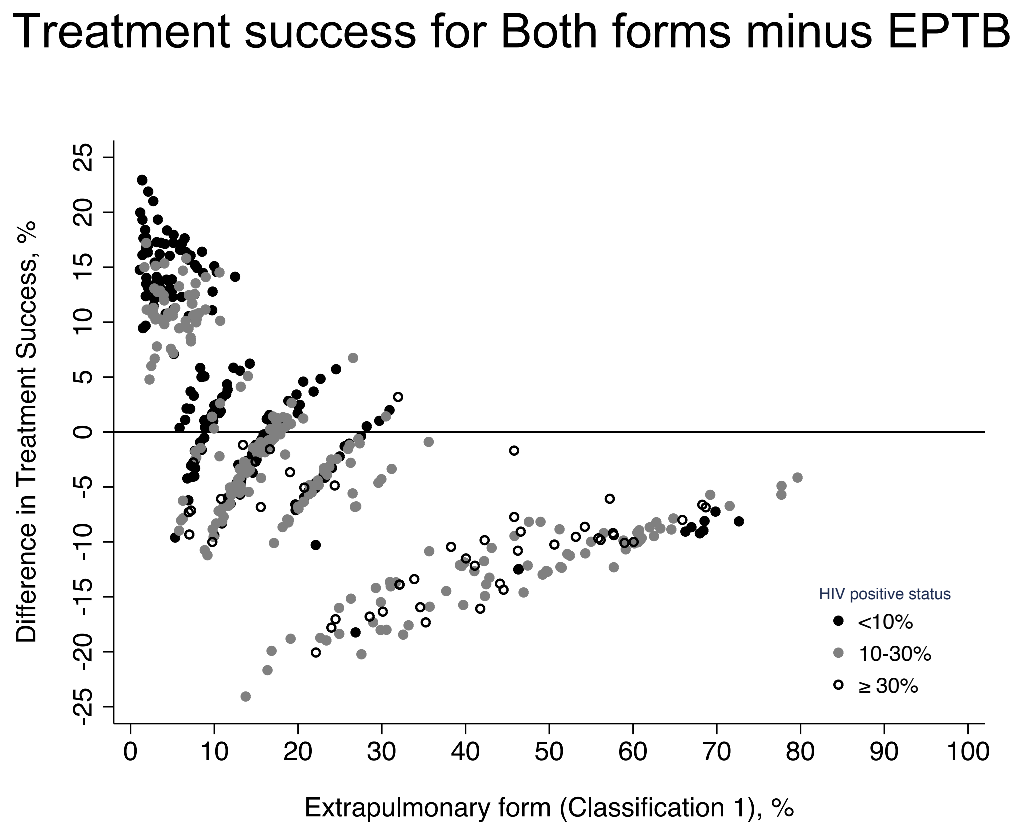

Difference in treatment success between overall (both forms) minus Extrapulmonary tuberculosis as classified by WHO (Classification 1).

EPTB denotes extrapulmonary tuberculosis; HIV denotes human immunodeficiency virus; WHO denotes World Health Organization

Figure C. **Over or under-estimation in tuberculosis treatment success at country-level for 500 simulated countries between overall (both forms) and each for of tuberculosis as classified by WHO (Classification1) after full adjustment for potential confounders.**

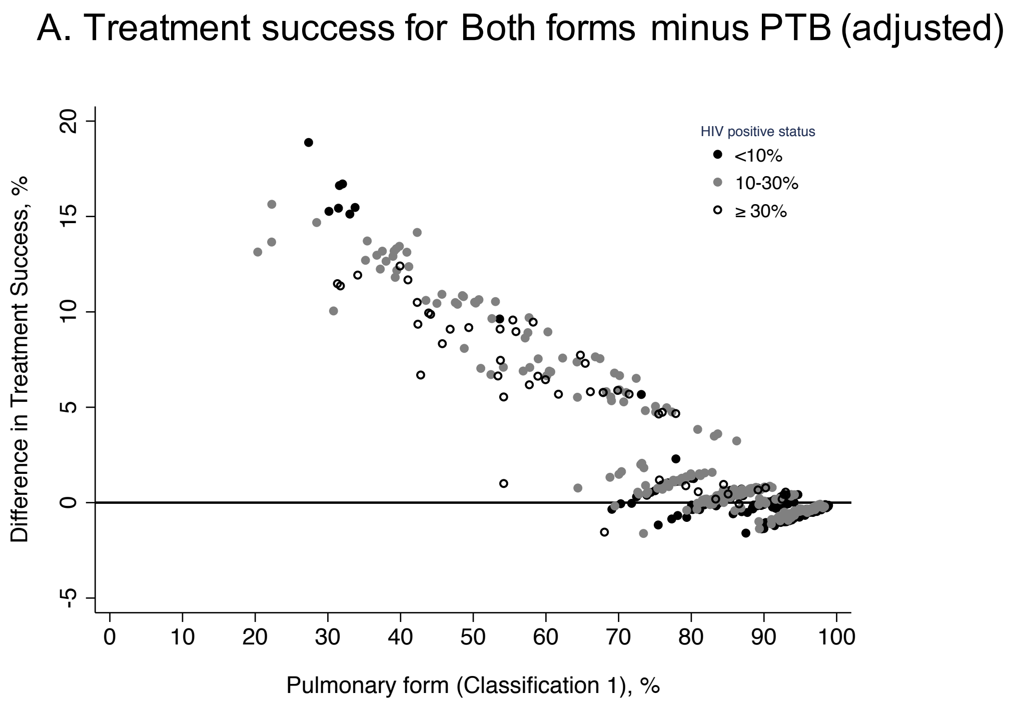

Panel A: Difference in treatment success between overall (both forms) minus Pulmonary tuberculosis as classified by WHO (Classification 1) after full adjustment for potential confounders.

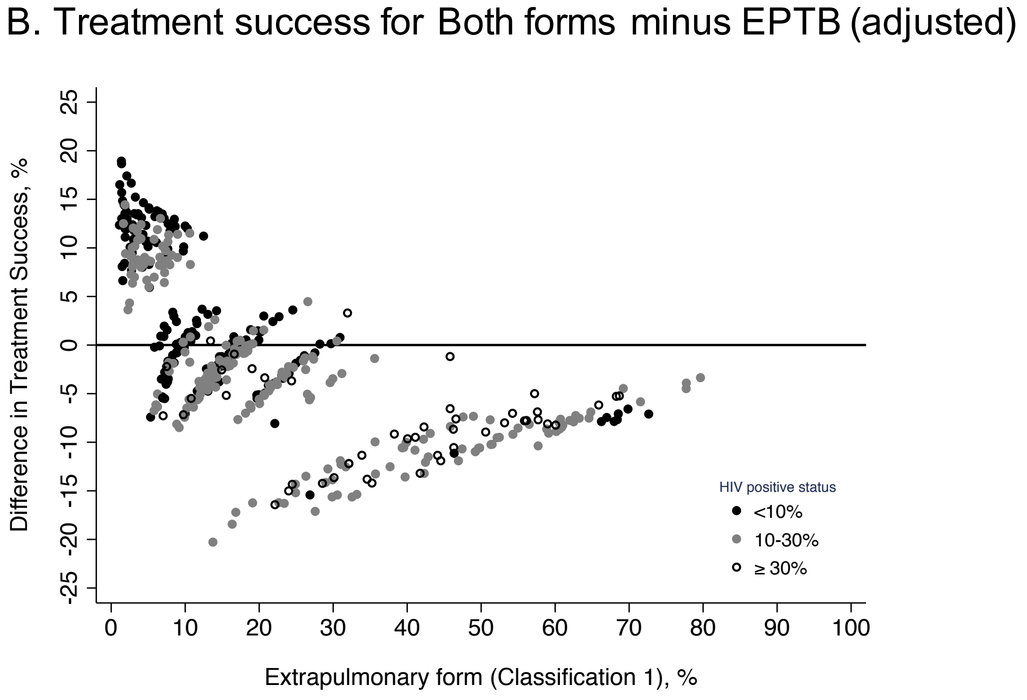

Panel B: Difference in treatment success between overall (both forms) minus Extrapulmonary tuberculosis as classified by WHO (Classification 1) after full adjustment for potential confounders.
